# Quantitative Detection of NADH Using a Novel Enzyme-Assisted Method Based on Surface-Enhanced Raman Scattering

**DOI:** 10.3390/s17040788

**Published:** 2017-04-07

**Authors:** Haiyan Teng, Mingyang Lv, Luo Liu, Xin Zhang, Yongmei Zhao, Zhenglong Wu, Haijun Xu

**Affiliations:** 1 Beijing Key Laboratory of Bioprocess, Beijing University of Chemical Technology, Beijing 100029, China; 15117955455@163.com (H.T.); lvmy1991@163.com (M.L.); liuluo@mail.buct.edu.cn (L.L.); 2 Engineering Research Center for Semiconductor Integrated Technology, Institute of Semiconductors, Chinese Academy of Sciences, Beijing 100083, China; ymzhao@semi.ac.cn; 3 Analytical and Testing Center, Beijing Normal University, Beijing 100875, China; wuzl@bnu.edu.cn

**Keywords:** surface enhanced Raman scattering, quantitative detection, NADH, enzyme-assisted

## Abstract

An enzymatic method for quantitative detection of the reduced form of nicotinamide-adenine dinucleotide (NADH) using surface-enhanced Raman scattering was developed. Under the action of NADH oxidase and horseradish peroxidase, NADH can generate hydrogen peroxide (H_2_O_2_) in a 1:1 molar ratio, and the H_2_O_2_ can oxidize a chromogen into pigment with a 1:1 molar ratio. Therefore, the concentration of NADH can be determined by detecting the generated pigment. In our experiments, eight chromogens were studied, and o-tolidine (OT) was selected because of the unique Raman peaks displayed by its corresponding pigment. The optimal OT concentration was 2 × 10^−3^ M, and this gave the best linear relationship and the widest linear range between the logarithmic H_2_O_2_ concentration and the logarithmic integrated SERS intensity of the peak centered at 1448 cm^−1^. Under this condition, the limit of detection for NADH was as low as 4 × 10^−7^ M. Two NADH samples with concentrations of 2 × 10^−4^ and 2 × 10^−5^ M were used to validate the linear relationship, and the logarithmic deviations were less than 3%.

## 1. Introduction

Nicotinamide adenine dinucleotide (NAD^+^) and its reduced form (NADH) are ubiquitous biomolecules found in eukaryotic and prokaryotic organisms. Both these compounds are key central charge carriers in living cells and are essential in energy metabolism, reductive biosynthesis, and antioxidation [1,2,3]. The NAD-linked dehydrogenases, a class of more than 300 enzymes, use this couple as coenzymes [4]. NADH plays key roles in cellular energy metabolism, such as ATP generation in mitochondria and biomass generation in fermentation processes [5,6,7]. Quantitative detection of NADH allows for understanding of the overall cellular energy metabolism and monitoring of fermentation processes. Several methods have been utilized to determine NADH, such as fluorescence spectra and electrochemical methods [8,9,10,11,12]. However, these methods are invasive, time consuming, and complex. Spectroscopic techniques may overcome these problems, and surface-enhanced Raman scattering (SERS) is particularly attractive.

SERS is an extremely sensitive technique that can be tailored to detect specific analytes using their unique vibrational fingerprints, and has been widely used in many fields, including physics, biology, environmental science, and chemistry [13,14,15,16,17,18]. The Raman intensity of a probe molecule can be enhanced by a factor of 10^3^–10^6^, and the narrow linewidth of SERS spectra allows for detection of multiple analytes in complex mixtures, even reaching single-molecule level [19,20,21]. The enhancement achieved with SERS can be primarily attributed to the electromagnetic mechanism [22,23], and gold (Au) and silver (Ag) are widely used as SERS-active substrates for their superior performances in supporting the electromagnetic enhancement. However, Au/Ag-based SERS substrates do not provide satisfactory detection of NADH. In early experiments, we found the characteristic peak of NADH centered at 1688 cm^−1^ disappeared when the concentration decreased to 10^−3^ M, and a NAD^+^ peak centered at 1032 cm^−1^ was observed (Figure S1) [4]. This may be due to the hydrogen ion of NADH being captured by Au or Ag, resulting in the generation of NAD^+^. Thus, there exists a huge obstacle in the direct detection of NADH on general SERS substrates. The enzyme-assisted method could be used to overcome this problem, and allow for application of simple Ag/Si substrates to the indirect detection of NADH.

The aim of this research was to develop a novel enzyme-assisted method based on SERS. The working principles of this method can be explained by the following equations.

(1) $$\mathrm{NADH}\;+\;H^{+}\;+\;O_{2}\overset{\mathrm{NOX}}{\to }{\mathrm{NAD}}^{+}{+H}_{2}O_{2}$$

(2) $$H_{2}O_{2}+\mathrm{Chromogen}\;\overset{\mathrm{HRP}}{\to }{\;H}_{2}O+\mathrm{Pigment}$$

NADH generates hydrogen peroxide (H_2_O_2_) with the action of NADH oxidase (NOX), and the H_2_O_2_ oxidizes a chromogen to a pigment with catalysis by horseradish peroxidase (HRP). The molar ratio of NADH, H_2_O_2_, and pigment is 1:1:1, and the concentration of NADH can be determined by quantifying the pigment. Eight chromogens were screened, and o-tolidine (OT) was selected as the most appropriate one instead of the commonly used 3,3′,5,5′-tetramethylbenzidine (TMB) [24]. Experiments were performed to determine the optimal concentration of OT, and 2 × 10^−3^ M was selected after SERS analysis. The linear relationship between the logarithmic concentration of H_2_O_2_ (lgC(H_2_O_2_)) and the logarithmic integrated SERS intensity of the peak centered at 1448 cm^−1^ (lgS_1448_) was established, and the correlation coefficient (R^2^) of the fitted curve was calculated to be 0.991. The limit of detection (LOD) of H_2_O_2_ was as low as 4 × 10^−7^ M, and this was the same for NADH. Two NADH solutions were used to verify the relationship curve, and the logarithm deviations of the detected values from the actual concentrations were less than 3%, indicating the detection method has good accuracy. To the best of our knowledge, no other studies have reported using OT as a chromogen in SERS detection, and the LOD of NADH reported in this paper is low. This enzyme-assisted method to detect NADH is sensitive and simple, and is promising for monitoring of fermentation processes and cell potential, and even for metabolism research.

## 2. Experimental Section

### 2.1. Materials and Instruments

OT, TMB, ABTS (2,2′-azinobis-(3-ethylbenzthiazoline-6-sulfonate)), BZ (benzidine), 4-CN (4-chloro-1-naphthol), DAB (3,3′-diaminobenzidine), 5-ASA (5-aminosalicylic acid), OPD (*o*-phenylenediamine), 30% wt H_2_O_2_, HRP, NADH, K_2_HPO_4_, and KH_2_PO_4_ were purchased from J&K Scientific Ltd. (Beijing, China). The NOX crude liquid was prepared by the Beijing Key Laboratory of Bioprocess. Deionized water (18 MΩ) supplied by Beijing Chemical Works was used for all experiments. All chemicals, unless otherwise stated, were of analytical grade and were used as received.

### 2.2. Sample Preparation

The Ag/Si substrate was prepared using the method described by Zhang et al. [25]. The synthesis was carried out at room temperature. N-type (100) Si wafers (1–10 Ω·cm) were used as the substrate material and cut into pieces (1 × 3 cm^2^). The cut Si wafer was cleaned sequentially in deionized water, acetone, and alcohol for 5 min each. The wafer was then immersed in a solution of 4.6 M HF and 0.005 M AgNO_3_ for 2 min and then washed several times with deionized water. Then, the template was placed in a mixture of 4.6 M HF and 0.5 M H_2_O_2_ for 60 min. Finally, the template was immersed in a solution of 0.01 M AgNO_3_ until the surface became milky white. The substrates were characterized by field emission scanning electron microscopy and transmission electron microscopy measurements (Figure S2).

### 2.3. SERS Measurements

All samples were measured at room temperature with a LabRAM ARAMIS Raman system equipped with 532, 633, and 785 nm lasers for excitation. For the detection of different types of chromogens and pigments, different excitation wavelengths were used to obtain the best Raman signals (Table S1). The excitation wavelength for the detection of OT and OT diimine was 785 nm because this gave the weakest background signal (Figure S3). The diameter of the light spot was approximately 1 μm and the spectral resolution was 2 cm^−1^. For all measurements, the laser power was 0.35 mW and the signal accumulation time was 15 s. All the data were averaged over 20 randomly selected positions.

## 3. Results and Discussion

### 3.1. Chromogen Screening

The principles of this method involved detecting the pigment by SERS, using this to quantify H_2_O_2_, and then calculating the concentration of NADH (C(NADH)). Therefore, we need to establish what was an appropriate chromogen and construct a curve for quantifying H_2_O_2_.

**Figure 1:**
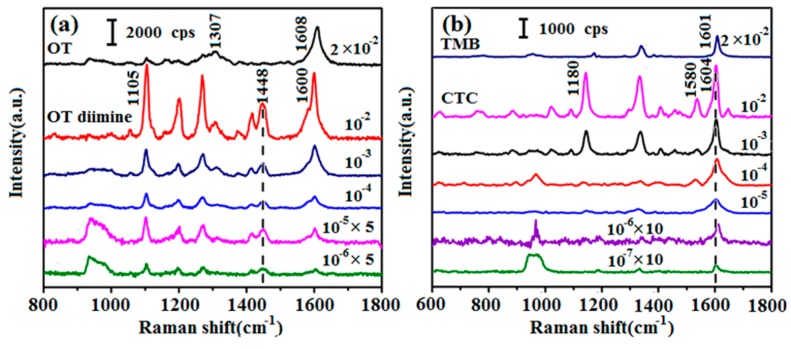
(**a**) Surface-enhanced Raman scattering (SERS) spectra of 2 × 10^−2^ M o-tolidine (OT), and OT diimine with different concentrations from 10^−2^ to 10^−6^ M; (**b**) SERS spectra of 2 × 10^−2^ M 3,3′,5,5′-tetramethylbenzidine (TMB) and charge transfer complex (CTC) with different concentrations from 10^−2^ to 10^−7^ M.

C(NADH) is unknown in actual detection, so an excess of the chromogen should be used to ensure complete reaction of H_2_O_2_ from the oxidation of NADH. However, the residual chromogen in the solution may produce strong Raman peaks, which could interfere with those of the pigment if they overlap or are close, making the quantification of the pigment difficult. Therefore, choosing an appropriate chromogen is crucial for NADH detection. The chromogen should display some strong characteristic Raman peaks that are distinguishable from those of the pigment. In this study, eight commonly used chromogens were screened by detailed SERS analyses. Using OT as an example, the experimental process involved taking 100 μL of OT (2 × 10^−2^ M to 2 × 10^−7^ M) in phosphate buffer (pH 8.0, 0.1 M) and mixing it with 100 μL of H_2_O_2_ (10^−1^ M to 10^−6^ M). Then, 1 mg of HRP was added and the solution was left at room temperature for 20 min so that the reaction could reach completion. OT and H_2_O_2_ reacted in a 1:1 ratio under these conditions, and excess H_2_O_2_ ensured that all of the chromogen was oxidized to pigment (Figure S4). Experiments with the seven other chromogens were carried out in the same way. SERS spectra were obtained of OT, TMB, and the corresponding pigments (Figure 1). The SERS spectra of the other six chromogens are shown in Figure S5. From these results, OT was selected as the chromogen for the detection of NADH. For OT (Figure 1a), the main Raman peaks were centered at 1104, 1307, and 1608 cm^−1^. Some unique peaks of OT diimine, which was the oxidation product of OT, were observed at 1105 and 1448 cm^−1^. Among these peaks, the one centered at 1448 cm^−1^, which was assigned to the C=N stretching band, was the characteristic peak of OT diimine [26], and its appearance indicated H_2_O_2_ had been consumed and the pigment had been generated. For 10^−2^ M TMB (Figure 1b) and its corresponding pigment charge transfer complex (CTC) in the range from 10^−2^ M to 10^−7^ M, some Raman peaks of CTC were strong and sharp but most of them overlapped with or were close to those of TMB. Moreover, the unique peaks of CTC, such as those at 1180 and 1580 cm^−1^, decreased irregularly and were difficult to distinguish at low concentrations, which would be a serious obstacle to the quantitative analysis of the enzyme reaction product by SERS [27]. The SERS spectra of the other chromogens (Figure S5) showed lower sensitivity or no characteristic peaks. Considering the need to avoid residual chromogen and the requirement for a low LOD, OT was selected for subsequent experiments.

### 3.2. Optimization of the OT Concentration

**Figure 2:**
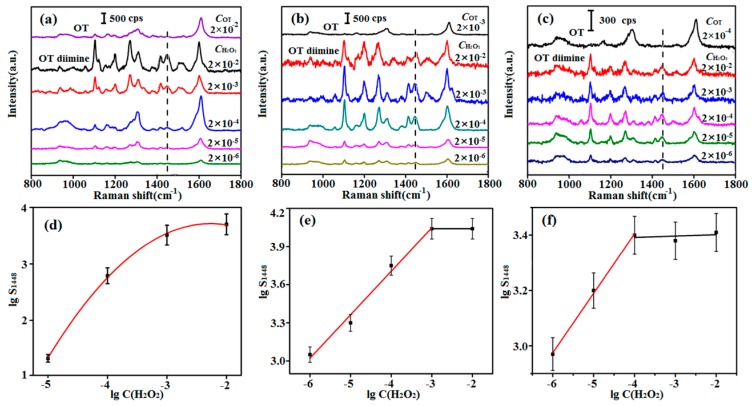
SERS spectra of (**a**) 2 × 10^−2^ M OT and OT diimine with different concentrations produced from the reaction of 2 × 10^−2^ M OT and 2 × 10^−2^ to 2 × 10^−7^ M H_2_O_2_; (**b**) 2 × 10^−3^ M OT and OT diimine with different concentrations produced from the reaction of 2 × 10^−3^ M OT and 2 × 10^−2^ to 2 × 10^−7^ M H_2_O_2_; (**c**) 2 × 10^−4^ M OT and OT diimine with different concentrations produced from the reaction of 2 × 10^−4^ M OT and 2 × 10^−2^ to 2 × 10^−7^ M H_2_O_2_; (**d**–**f**) show the dependence of the lgS_1448_ on lgC(H_2_O_2_); corresponding to (**a**–**c**), respectively.

As mentioned above, the chromogen must be excess to ensure the complete reaction of H_2_O_2_ in actual detection. Therefore, the OT concentration in the solution should be optimized. Considering that C(NADH) in organisms usually alters in the range of 10^−4^~10^−6^ M, OT with certain concentrations of 2 × 10^−2^, 2 × 10^−3^, and 2 × 10^−4^ M were selected to react with different concentrations of H_2_O_2_ (2 × 10^−2^ to 2 × 10^−7^ M), respectively. HRP was used as the catalyst and the reaction conditions were the same with those in the chromogen screening process. Due to the increase of solution volume, C(OT) and C(H_2_O_2_) were diluted to be a half of the original concentration. And the mole ratio of H_2_O_2_ and OT diimine was 1:1 as shown in Figure S4, the concentration of OT diimine can be calculated. The SERS test results (Figure 2a–c), together with curves showing the relationship between lgS_1448_ and lgC(H_2_O_2_) (Figure 2d–f), showed that the relationship curve was not linear when the OT concentration was 2 × 10^−2^ M. Linear regions were observed for 2 × 10^−3^ and 2 × 10^−4^ M OT. The linear range of 2 × 10^−3^ M OT was 10^−3^ to 10^−6^ M, and was wider than that of 2 × 10^−4^ M OT (10^−4^ to 10^−6^ M). Consequently, it was selected for reaction with H_2_O_2_ in subsequent experiments. It is worth noting that the OT diimine concentration remained constant with excess H_2_O_2_ (Figure 2e,f), further explaining that OT and H_2_O_2_ reacted as 1:1.

### 3.3. Establishment of the Relationship Curve

**Figure 3:**
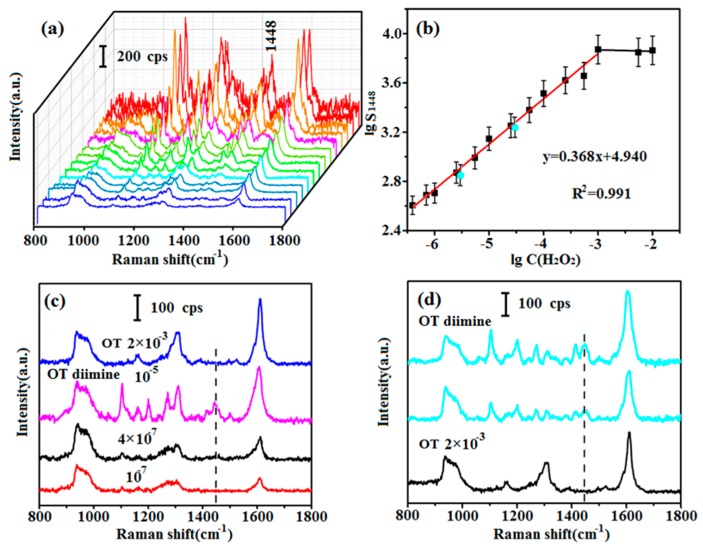
(**a**) SERS spectra of OT diimine obtained with different concentrations of H_2_O_2_; (**b**) relationship curve between lgS_1448_ and lgC(H_2_O_2_), with validation data shown in blue dots; (**c**) SERS spectra of the 2 × 10^−3^ M OT, and the 10^−5^, 4 × 10^−7^, 10^−7^ M OT diimine. The 1448 cm^−1^ peak marked with the dotted line is the characteristic peak; (**d**) SERS spectra of OT diimine for validation, and that of 2 × 10^−3^ M OT.

For quantitative detection of NADH, an accurate curve showing the relationship between C(H_2_O_2_) and S_1448_ of OT diimine should be established. In addition to the 2 × 10^−2^ to 2 × 10^−6^ M experiments (Section 3.2), experiments were conducted with H_2_O_2_ concentrations of 1.1 × 10^−2^, 1.1 × 10^−3^, 5 × 10^−4^, 1.1 × 10^−4^, 5 × 10^−5^, 1.1 × 10^−5^, 5 × 10^−6^, 1.4 × 10^−6^ and 8 × 10^−7^ M. The H_2_O_2_ was reacted with 2 × 10^−3^ M OT, and SERS spectra of the reaction products were recorded (Figure 3a). The intensity of the peak centered at 1448 cm^−1^ clearly decreased as the concentration of OT diimine decreased except the strongest three, which were corresponding to the excess H_2_O_2_ with concentrations of 2 × 10^−2^ M, 1.1 × 10^−2^ M and 2 × 10^−3^ M, respectively. Based on the experimental results, a curve illustrating this relationship was established (Figure 3b). Obviously, C(H_2_O_2_) and S_1448_ showed a good linear relationship under the logarithmic coordinate except the highest three points corresponding to the strongest three spectra in Figure 3a. The linear range from 10^−3^ M to 4 × 10^−7^ M was described by the equation lgS_1448_ = 0.368 lgC(H_2_O_2_) + 4.940, and the correlation coefficient was *R*^2^ = 0.991. Thus, C(H_2_O_2_) can be determined using the SERS spectra of OT diimine. The LOD of OT diimine was also determined. OT does not give a 1448 cm^−1^ Raman signal, and the presence of this signal indicates the production of OT diimine in solution. The 1448 cm^−1^ Raman signal was detected when with 4 × 10^−7^ M OT diimine, but disappeared at 10^−7^ M OT diimine (Figure 3c). Therefore, the LOD of OT diimine was 4 × 10^−7^ M. The LOD of H_2_O_2_ would also be 4 × 10^−7^ M.

### 3.4. Validation of the Relationship Curve

To validate the detection system, two verifying detection were done. NADH (100 μL, 2 × 10^−4^ or 2 × 10^−5^ M) was injected into a solution containing 100 μL of OT (2 × 10^−3^ M). Next, 500 μL of NOX and 1 mg of HRP were added, and the mixture was gently shaken for 30 min to ensure complete reaction of NADH. Because of the addition of OT and NOX, the actual C(NADH) in the solution was diluted to 2.86 × 10^−5^ or 2.86 × 10^−6^ M, which corresponded to −4.544 and −5.544 of lgC(NADH) respectively. SERS spectra of the final reaction products and 2 × 10^−3^ M OT were obtained (Figure 3d). A peak centered at 1448 cm^−1^ was clearly observed, which indicated that OT diimine was generated and NADH was consumed. The S_1448_ values are 1722 and 706, respectively, corresponding to the blue points in Figure 3b. The lgS_1448_ are 3.236 and 2.849 respectively, and the lgC(H_2_O_2_) values can be calculated to be −4.630 and −5.682 according to the established mathematical equation. Because the molar ratio of NADH to H_2_O_2_ is 1:1, the calculated lgC(NADH) should be −4.630 and −5.682 respectively, corresponding to 4.084 × 10^−5^ and 1.774 × 10^−6^ M of C(NADH). When the actual and calculated C(NADH) in solution were compared (Table 1), the logarithm deviations were 1.98% and 2.53%. Therefore, this enzyme-assisted method is feasible for quantitative detection of NADH.

## 4. Conclusions

Quantitative detection of NADH can be realized using SERS spectra of the reaction product of NADH after enzyme catalysis. OT, which is rarely used in SERS detection, is a better chromogen for this application than TMB or other common chromogens. The optimum OT concentration is 2 × 10^−3^ M, and this gives better linearity and a wider linear range than other concentrations. The linear relationship between lgC(H_2_O_2_) and lgS_1448_ can be used to quantify C(NADH). This enzyme-assisted method for NADH detection does not rely on the Raman intensity of NADH, and the LOD is of the same magnitude as that for the pigment (i.e., as low as 4 × 10^−7^ M). Besides, it is worth mentioning that higher sensitivity might be further expected if we could find a more appropriate chromogen. This enzyme-assisted method for NADH detection and the discovery of the unique advantages of OT for SERS analysis can be used to monitor biomass generation in fermentation processes. Additionally, it provides some useful inspiration for the detection of unstable substances with weak Raman signals in the future.

## Figures and Tables

**Table 1:** Comparison of the actual and the measured concentrations of NADH.

Sample	S_1448_	lgC(NADH)		RSD (％)	Ζ (％)
		Actual	Calculated		
1	1722	−4.54	−4.63	2.42	1.98
2	706	−5.54	−5.68	2.09	2.53

## Supplementary Materials

The following are available online at http://www.mdpi.com/1424-8220/17/4/788/s1, Figure S1: (a) SERS spectra of NADH on the Au-based SERS substrate; (b) SERS spectra of NADH on the Ag-based SERS substrate. Figure S2: (a) Top, (b) magnified, (c) cross-sectional FE-SEM and (d) TEM images of Ag/Si substrate. Figure S3: SERS spectra of OT diimine tested on the Ag/Si substrate with different excitation wavelengths (532, 633 and 785 nm). Figure S4: Horseradish peroxidase (HRP) catalyzed oxidation of OT by H_2_O_2_. One molecule OT is oxidized by one molecule H_2_O_2_ and forms one molecule OT diimine. Figure S5: SERS spectra of (a) ABTS and the corresponding pigment, (b) BZ and the corresponding pigment, (c) 4-CN and the corresponding pigment, (d) DAB and the corresponding pigment, (e) ASA and the corresponding pigment, and (f) OPD and the corresponding pigment. The concentration of each sample is marked. Table S1: Detailed information of the SERS test of eight types of chromogen.
