# Supplementary material for: Quantitative Detection of NADH Using a Novel Enzyme-Assisted Method Based on Surface-Enhanced Raman Scattering
Source: Sensors (Basel). 2017 Apr 7;17(4):788. doi: 10.3390/s17040788 (PMC5422061; doi:10.3390/s17040788)
Supplement: Supplementary file 1 [file sensors-17-00788-s001.pdf]

Supplemental Material

Quantitative Detection of NADH Using a Novel Enzyme-Assisted Method Based on Surface-Enhanced Raman Scattering

Haiyan Teng [1], Mingyang Lv [1], Luo Liu [1], Xin Zhang [1,*], Yongmei Zhao [2], Zhenglong Wu [3] and Haijun Xu [1,*]

[1] Beijing Key Laboratory of Bioprocess, Beijing University of Chemical Technology, Beijing, 100029, China; zhxin@mail.buct.edu.cn (X.Z.); hjxu@mail.buct.edu.cn (H.X.)

[2] Engineering Research Center for Semiconductor Integrated Technology, Institute of Semiconductors, Chinese Academy of Sciences, Beijing, 100083, China; ymzhao@semi.ac.cn (Y.Z.)

[3] Analytical and Testing Center, Beijing Normal University, Beijing 100875, China；wuzl@bnu.edu.cn (Z.W.)

* Correspondence: zhxin@mail.buct.edu.cn; hjxu@mail.buct.edu.cn; Tel.: +86-10-64442357; Fax: +86-10-64435170

The results of detecting NADH on the Au/Ag-based SERS substrates. According to our previous experiments, the characteristic peak of NADH centered at 1688 cm^{-1} disappeared when the concentration decreased to 10^{-3} M on the Au-based SERS substrate. Meanwhile, a peak of NAD^{+} centered at 1032 cm^{-1} was observed as shown in Figure S1(a). And the result of Ag-based SERS substrate is similar to Au-based SERS substrate as shown in Figure S1(b).

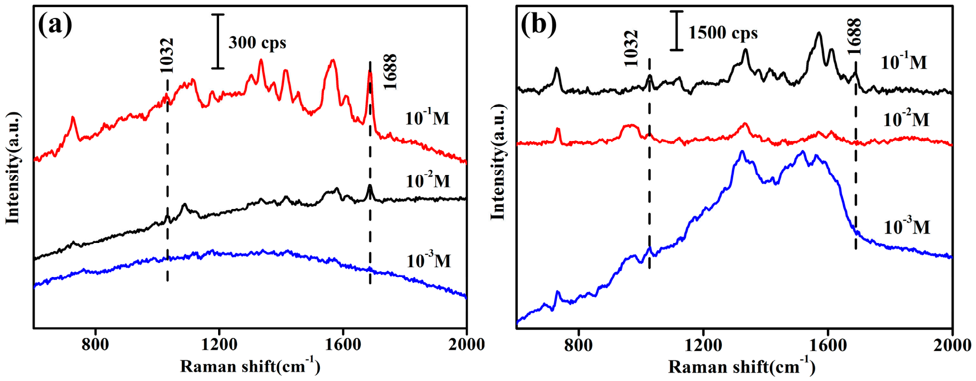

Figure S1. (**a**) SERS spectra of NADH on the Au-based SERS substrate. (**b**) SERS spectra of NADH on the Ag-based SERS substrate.

Characterization of Ag/Si substrate. Figure S2(a) is a top FE-SEM image of the substrate, which shows clearly that a significant amount of Ag nanoparticles (NPs) with an average diameter of 400 nm were deposited on the Si nanowire (NW) array surface. The amplified image shows that Ag NPs mainly adhered to the upper end of each Si NW as shown in Figure S2(b). Figure S2(c) is a cross-section image of Ag/Si substrate, which reveals that the length and diameter of Si NWs are 13 μm and 450 nm, respectively. Figure S2(d) is a TEM image of the substrate, which shows clearly that Ag NPs on the side of Si NW are small and sparse.

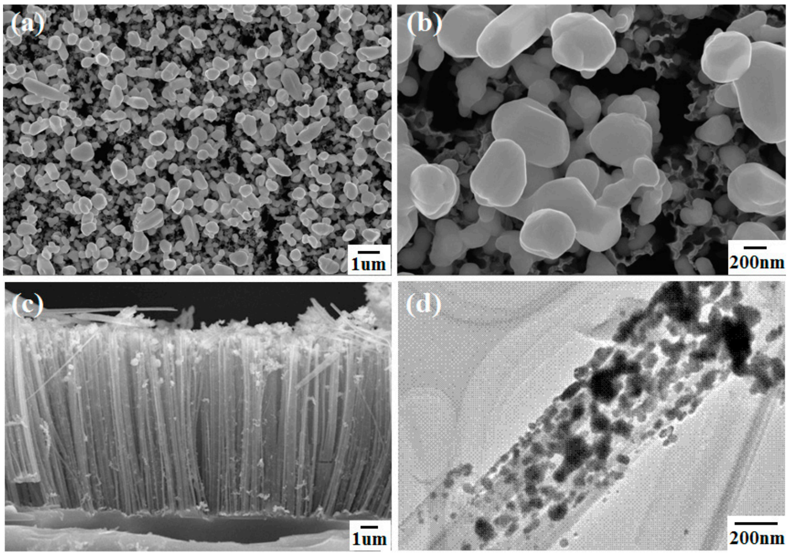

Figure S2. (**a**) Top, (**b**) magnified, (**c**) cross-sectional FE-SEM and (**d**) TEM images of Ag/Si substrate.

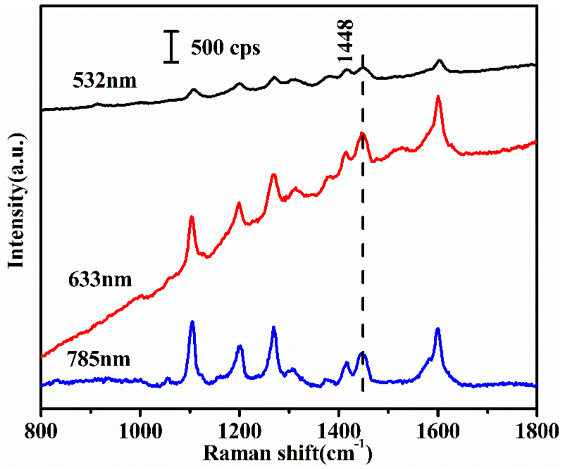

Figure S3. SERS spectra of OT diimine tested on the Ag/Si substrate with different excitation wavelengths (532, 633 and 785 nm).

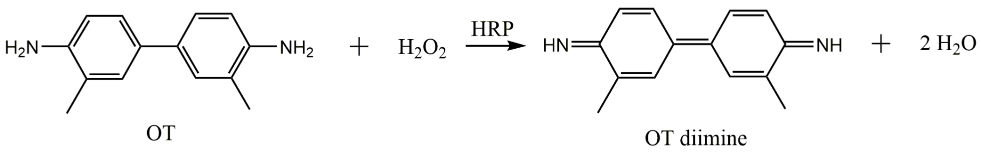

Figure S4. Horseradish peroxidase (HRP) catalyzed oxidation of OT by H_2O_2. One molecule OT is oxidized by one molecule H_2O_2 and forms one molecule OT diimine.

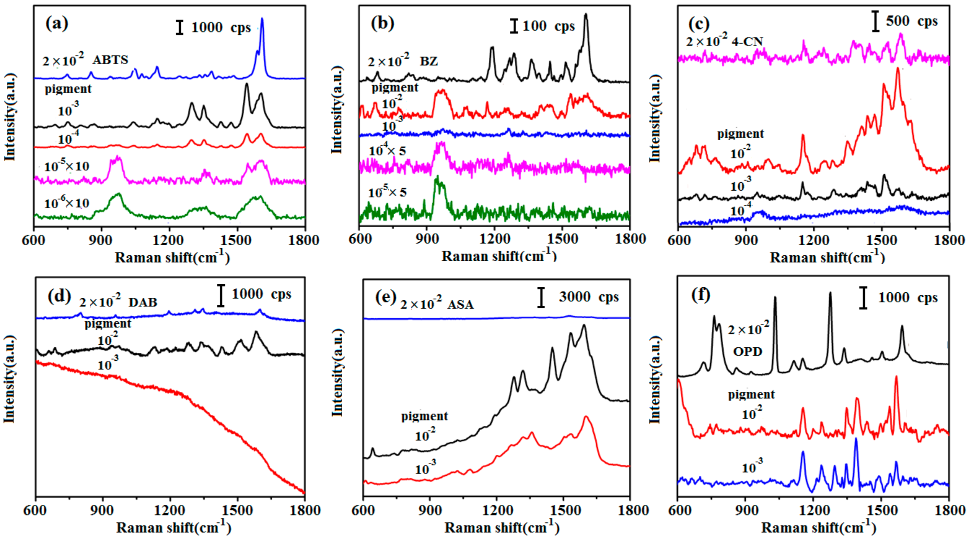

Figure S5. SERS spectra of (**a**) ABTS and the corresponding pigment, (**b**) BZ and the corresponding pigment, (**c**) 4-CN and the corresponding pigment, (**d**) DAB and the corresponding pigment, (**e**) ASA and the corresponding pigment, and (**f**) OPD and the corresponding pigment. The concentration of each sample is marked.

Table S1. Detailed information of the SERS test of eight types of chromogen.

Chromogen	Manufacturer	Purity	Excitation wavelength(nm)	LOD of Pigment (M)	SERS Peaks (cm^{-1})
3,3',5,5'-tetramethyl (TMB)	J&K	AR	633	10^{-7}	1604<br>1537<br>1334<br>1143
o-tolidine (OT)	J&K	AR	785	10^{-6}	1600<br>1448<br>1415<br>1105
[2,2'-azino-di(3-ethylbenzthiazoline-6-sulphonic acid) -2NH4-salt (ABTS)	J&K	AR	532	10^{-6}	1604<br>1539<br>1353<br>1298
Benzidine (BZ)	J&K	AR	633	10^{-4}	1254
4-chloro-1-naphthol (4-CN)	J&K	AR	633	10^{-3}	1628<br>1571<br>1153<br>713
3,3'-diaminobenzidine (DAB)	J&K	AR	785	N	1583<br>1512<br>1430<br>1281
5-aminosalicylic acid (5-ASA)	J&K	AR	633	N	1594<br>1531
O-Phenylenediamine (OPD)	J&K	AR	532	N	1583<br>1430<br>1281<br>1128

N:None
